# The new practice of interviews focusing on presenteeism provides additional opportunities to find occupational health issues

**DOI:** 10.1539/eohp.2021-0021-GP

**Published:** 2022-03-11

**Authors:** Kosuke Sakai, Tomohisa Nagata, Masako Nagata, Yoshihisa Fujino, Koji Mori

**Affiliations:** 1 Department of Occupational Health Practice and Management, Institute of Industrial Ecological Sciences, University of Occupational and Environmental Health, Japan, Kitakyushu, Japan; 2 Department of Environmental Epidemiology, Institute of Industrial Ecological Sciences, University of Occupational and Environmental Health, Japan, Kitakyushu, Japan

**Keywords:** efficiency, interview, presenteeism, task performance and analysis

## Abstract

**Objectives:**

Presenteeism refers to the condition of working while having health problems and can be one of the perspectives to assess the incompatibility between workers and their jobs. The purpose of this survey was to find out what kind of occupational health issues can be detected by occupational physicians’ interviews focusing on presenteeism.

**Methods:**

We conducted interviews with workers suffering from presenteeism in a food manufacturing company. The Work Functioning impairment scale (WFun) was used as the indicator of presenteeism. We discussed the occupational health issues and the necessity of additional interventions.

**Results:**

Thirty-nine workers with WFun score of 21 or higher were interviewed, and we have found nine cases in need of support. The workplace issues were structured into four categories: (i) health problems that are difficult to identify through health checkups, (ii) health problems missed by the stress check program, (iii) health problems caused by workload that cannot be identified by workplace patrols, and (iv) health problems that are not considered because they do not require support.

**Conclusions:**

We discovered new workplace issues by interviewing workers suffering from presenteeism.

## Background and Issue

The purpose of occupational health is to improve the adaptation of workers and their work. This philosophy is based on the goal described by the Joint International Labor Organization/World Health Organization Committee on Industrial Hygiene report in 1950: “the adaptation of work to man and of each man to his job”^1)^. To achieve this purpose, occupational health practitioners examine workplace issues in the adaptation using a variety of approaches, including interviews with occupational physician after health checkups, doctors’ interviews based on workers’ stress check programs, workplace patrols, and return-to-work planning after long-term sickness absence. However, each of these approaches involves certain limitations in identifying problems. For example, the kinds of health problems tested in general health checkups are limited and stressed workers may be reserved and fail to seek help voluntarily. Therefore, it is important for occupational health practitioners to identify the existing problems by combining various practices and to consider additional approach if the solution to these problems can be expected.

### Viewpoint on improvement

Presenteeism has recently been used as an indicator to investigate health-related workplace issues^2)^. It is defined as “attending work while ill,” in which employees continue working even when they are experiencing health problems or symptoms that affect their work performance^3)^. In particular, this indicator has been used in group analysis^4)^. One previous study reported that musculoskeletal pain and psychiatric disorders cause a greater loss of productivity through presenteeism compared with other health problems^5)^. As described above, presenteeism scales have been used to evaluate productivity loss at the group level, but not used to evaluate functional impairment at the individual worker level^6)^. Employees working while sick are likely to have maladaptation with their jobs. Understanding the problems underlying presenteeism could contribute to the development of new approaches in occupational health.

The purpose of the current survey was to describe the occupational health issues observed in interviews for individual workers with presenteeism.

## Implementation

### Features of the workplace

We conducted this survey in the headquarters and main factory of one food company. There were 921 full-time and part-time workers in these workplaces. They were in the administrative departments, the manufacturing department, and the logistics department. Some permanent night shift workers were employed in the manufacturing department.

### Selection of interviewees based on presenteeism

We selected subjects using a presenteeism questionnaire and then conducted an interview survey. Prior to the selection procedure, we developed a questionnaire that included the Work Functioning Impairment Scale (WFun)^7)^. Workers were required to complete the questionnaire during a health checkup conducted in November and December 2020. The inclusion criteria of this interview survey were workers who scored 21 points or more on the WFun, which indicated moderate or high work functional impairment^8)^, and not having obvious health problems in health checkups requiring an interview with an occupational physician to consider work restrictions.

**Fig. 1.**
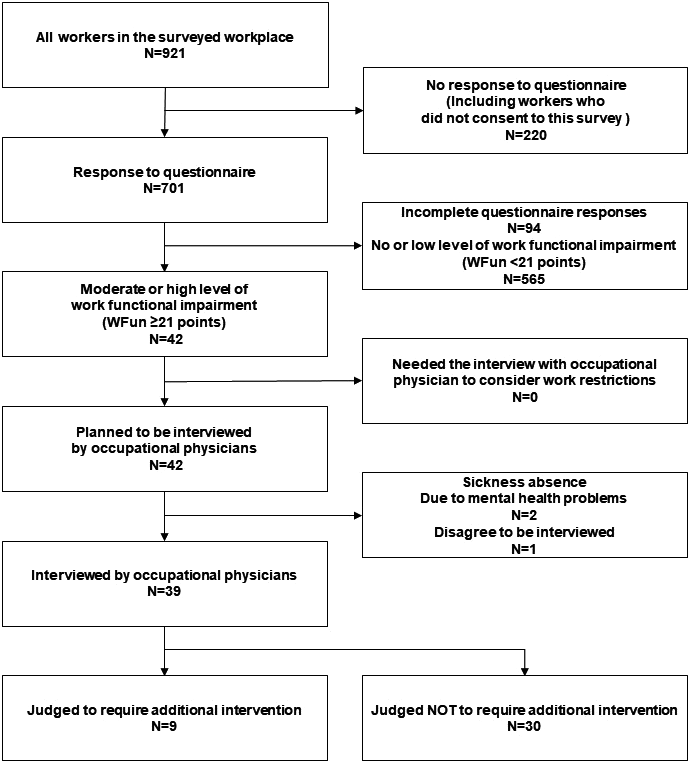
The flow diagram of presenteeism interviewees.

The total of 921 workers underwent health checkups, and 701 workers agreed to participate in the study and completed the questionnaire. We then excluded 94 workers who had incomplete questionnaires and 565 workers whose WFun score was less than 21 points. There were no workers with health problems that were judged to require an interview with occupational physician to consider work restrictions. We excluded 3 workers who were supposed to be interviewees in the plan; 2 were on leave due to mental health issues, and 1 did not give consent to participate in the interview. Ultimately, we included 39 workers (29 men) in the interview survey. After the interviews, nine of the interviewees were identified as needing additional intervention by their occupational physicians (Figure 1).

### Survey regarding individual and workplace causes of presenteeism

The interviews were conducted by two part-time occupational physicians in the company (KS and KM, among the authors) who understood the work conditions at the surveyed workplaces through regular occupational health activities, such as workplace patrols and participation in health and safety meetings.

The interviewers were two male occupational physicians: KM is 61 years old and has been an occupational physician as a specialist for about 30 years; KS is 30 years old and has been an occupational physician as a trainee for 3 years. Before the interviews, we created an interview guide consisting of the following sections: job description, presence of physical and psychological symptoms, time course, impact on work and life, location of causes, coping behaviors, prospects for improvement, and consultation history with managers and others. From January 2020 to March 2021, the occupational physicians conducted interviews with the workers based on the interview guide for 20 minutes per worker. They conducted the first three interviews together to unify the interview method, and then each of them made the other interviews separately.

Based on the content of each interview, the occupational physicians determined whether additional interventions were necessary. When KS was unable to decide whether additional action was needed, he consulted with KM. Because the resources of occupational health practices are limited, we thought that additional intervention from the occupational physician was unnecessary when the workers in presenteeism were able to cope with the problem by themselves, or when they were supported by their supervisor or medical doctor appropriately. Considering the above, we determined in all cases whether additional intervention from the occupational physician or the supervisor was necessary.

### Ethics

The implementation of this survey was discussed at a health and safety meeting in the workplace. We prepared an explanatory document about this survey and provided it to interviewees before the questionnaire. We explained that we would conduct the interviews with the aim of finding out what workplace issues were behind the work-related functional impairment. Only those who were able to provide consent were included in the study. After we wrote the paper, we obtained confirmation of the content and consent for publication for the nine interviewees described in detail.

This survey was approved by the ethics committee of the University of Occupational and Environmental Health, Japan (R1-058).

## Effect and outcome

### Characteristics of the participants in the survey

Table 1 shows the difference in characteristics between 39 interviewees (WFun ≥21) and 565 non-interviewees (WFun <21). The interviewees were employed full-time (66.7% vs. 45.5%), having some subjective symptoms (79.5% vs. 51.3%), current smokers (30.8% vs. 25.0%), and obese (with a BMI of 25 or higher; 30.8% vs. 23.9%).

**Table 1.** The characteristics of interviewees (WFun ≥21) and Non-interviewees (WFun <21)

		Interviewees (WFun≥21)		NON interviewees (WFun<21)	
		N	%	N	%
Total number of workers		39	100.0	565	100.0
Gender					
	women	11	28.2	274	48.5
	men	28	71.8	291	51.5
Age, years					
	≤29	7	17.9	46	8.1
	30–39	10	25.6	78	13.8
	40–49	11	28.2	176	31.2
	50–59	8	20.5	173	30.6
	≥60	3	7.7	92	16.3
Employment type					
	full-time	26	66.7	257	45.5
	part-time	13	33.3	308	54.5
WFun, points					
	0–6			42	7.4
	7–14			455	80.5
	15–20			68	12.0
	21–27	34	87.2		
	28–35	5	12.8		
Subjective symptoms					
	yes	31	79.5	290	51.3
Medical History					
	cerebral hemorrhage or stroke	0	0.0	7	1.2
	myocardial infarction or angina pectoris	0	0.0	7	1.2
	chronic renal failure, Hemodialysis	0	0.0	3	0.5
History of treatment with medication					
	hypertension	4	10.3	90	15.9
	diabetes mellitus	0	0.0	14	2.5
	dyslipidemia	0	0.0	59	10.4
Smoking status					
	current	12	30.8	141	25.0
	never or cessation	27	69.2	424	75.0
Drinking status					
	everyday	7	17.9	128	22.7
	sometimes	12	30.8	149	26.4
	rarely drink (cannot drink)	20	51.3	288	51.0
Body mass index, kg/m2					
	<20	9	23.1	135	23.9
	20≤, <25	18	46.2	295	52.2
	25≤, <30	8	20.5	95	16.8
	30≤	4	10.3	40	7.1

WFun, Work Functioning Impairment Scale.

### Cases in which additional intervention was required (9 workers)

Table 2 shows the interview content and judgments of nine cases in which the occupational physician determined that additional interventions were necessary after the interview. We discussed why the cases had not been identified in existing occupational health practices, such as health checkups, stress check programs, workplace patrols, and voluntary health consultations from workers. We realized that we had found health problems that had been overlooked due to deficiencies in existing occupational health practices, and we structured them into four categories:

**Table 2.** Cases determined that individual or organizational actions were needed

Case	Sex	Age	WFun	Details of the interview	Judgment of the interviewer	Category
1	man	33	31	He was obese and have been told that he snore by his family member. He worked on a production line and order supplies. He often made mistakes in ordering, and was often blamed for his mistakes.	It was necessary to recommend seeing a doctor for suspected obstructive sleep apnea.	i
2	man	36	35	His boss often got angry with him if he could not work quickly enough to keep up with the production line. His boss had even told him “I will knock you down”. He had difficulty falling asleep and woke up many times during the night because of work-related frustration.	The facts of the harassment needed to be confirmed and the impact on colleagues needed to be investigated.	ii
3	woman	61	24	One year has passed since she started working at the factory. Because she was not provided with sufficient training to become skilled, She became sluggish on the production line and her coworkers became angry with her. This increased the burden on her coworkers and interfered with her ability to build relationships with them.	If sufficient training opportunities were not provided to new employees, it would cause a problem for securing human resources. It was necessary to check and review the education system.	ii
4	man	46	28	He was a manager with a heavy workload and his colleages expected too much of him. He was losing confidence in his abilities in his work role. He had mid-waking and low back pain caused by a herniated disc.	Workplace patrols did not enable us to identify the difficulties that managers and people in other positions of responsibility had in their work. Mental health support was needed.	ii
5	man	54	24	He was aware of musculoskeletal pain in his neck, shoulders, lumbar region, and his whole body caused by the heavy work of repeatedly feeding the dough. His symptoms became stronger when he was immobile for a while or when it was cold in the plant.	Because the occupational physician was not aware of the work during the usual workplace patrol, it was necessary to check the work site to assess the physical effects.	iii
6	man	44	25	He have had acute back pain three times when lifting heavy objects. He felt anxious about the pain recurring before going to work and during work.	It was difficult to ascertain workers’ feelings of impatience and anxiety during workplace patrols. It was necessary to provide psychological support and improve the work environment to prevent the recurrence of back pain.	iii
7	man	56	24	He had been suffering from low back pain for the past year, which is related to heavy lifting. He was short and had to work on his toes. In addition, he was aware of the decline in his physical strength caused by aging. His doctor pointed out that he had a herniated disc, which led him to take a leave of absence.	The worker was aware of their physical maladjustment to the work, including their age and height, but continued to work without seeking support, which worsened the situation. It would be necessary to consider an appropriate work environment when he returns to work.	iv
8	man	23	22	Since childhood, He had suffered from unexplained headaches. He had to interrupt his work to take medication when the symptoms appear. His colleagues pointed out that he was unsociable.	Reasonable consideration should have been given to interrupting work during headache onset, and to ensure rest periods.	iv
9	man	44	25	He had a hearing impairment caused by a sudden hearing loss in the past that has not improved. He had trouble communicating with his colleagues in a noisy workplace.	There was a need to consider changing workplaces to enable employees to work more comfortably.	iv

i) Health problems that are difficult to identify through health checkups

ii) Health problems missed by the stress check program

iii) Health problems caused by workload that cannot be identified by workplace patrols

iv) Health problems that are not considered because they do not require support

#### 1. Health problems that are difficult to identify through health checkups

Health problems that were difficult to identify in regular health checkups were revealed through presenteeism interviews.

*In case 1, sleep apnea syndrome was suspected because of obesity, snoring during sleep, and a perceived loss of sound sleep. We recommended a medical examination for sleep apnea syndrome.*

Although it was possible to add questions about sleep disorders to the health screening questionnaire, there are other health problems that may also cause occupational dysfunction, and it is difficult to review all diseases during health checkups. Therefore, the measure of presenteeism may be useful for identifying a wide range of health problems.

#### 2. Health problems missed by the stress check program

In some cases, problems caused by psychological stress, such as with interpersonal relationships in the workplace, were revealed as the cause of presenteeism.

*In case 2, the worker was harassed by a supervisor when work was delayed on a production line. We reported the harassment to the supervisor and discussed countermeasures.*

*In case 3, the worker fell behind in learning necessary skills, which increased the burden for her colleagues. Eventually, she was isolated from them. We advised the manager to enhance training opportunities.*

*In case 4, the worker was experiencing psychological stress related to being in a position of responsibility. We advised him to take a temporary sick leave to care for his sleep disturbances and depressive symptoms.*

In the recent stress check program, conducted between May and July 2020, the workers (Cases 2, 3, and 4) were assessed as highly stressed, but they did not request any interviews with the occupational physician. The stress check program is designed in such a way that even if workers are found to be under high stress, they are not given the opportunity to meet with an occupational physician unless they proactively seek help. In this survey, we were able to contact stressed workers who had not sought help in the stress check program.

#### 3. Health problems caused by workload that cannot be easily identified by workplace patrols

We were able to understand the problems caused by workload, which are not easy to grasp through usual workplace patrols.

*In case 5, the worker had been suffering from pain in his neck, shoulders, and back caused by long hours of continuous work. We taught him self-care for his back pain and provided psychological support to him.*

*In case 6, the worker experienced repeated bouts of acute low back pain from heavy lifting, which led to chronic low back pain. We patrolled his workplace and advised the supervisor to improve the work environment to prevent recurrence of back pain.*

During workplace patrols, occupational health practitioners can ascertain the work environment and the tasks performed, but it may be difficult to evaluate all the physical adverse effects of non-routine tasks or repetitive small tasks. The interviews enabled us to identify problems that could not be found during workplace patrols.

#### 4. Health problems that are not considered because they do not require support

We were able to identify workers who were working without any support because their health problems had not worsened to the point of causing a leave of absence.

*In case 7, the worker was engaged in physical labor and suffered from a lumbar disc herniation. After the questionnaire, the worker’s pain worsened, and he had to take a period of sick leave. When he returned to work, we supported him to reduce heavy lifting to prevent recurrence.*

*In case 8, the worker had been aware of unexplained headaches since he was a child. He had difficulty with continuous work at the factory. In addition, co-workers around him did not understand him. As a rational consideration for him, we proposed to suspend work to take headache medication.*

*In case 9, the worker was in a noisy office and suffered from hearing loss, which made it difficult for him to communicate with his coworkers. We suggested to the manager to consider a change in his job description.*

When workers seek consultation regarding difficulties in the work environment, occupational physicians can advise them to change their workplaces or improve their work environment. In some cases, occupational physicians provide return-to-work support programs after long-term absences. However, some workers with health problems were unable to access these support measures and were working in inappropriate environments.

### Cases in which additional intervention was NOT required (30 workers)

We determined that 30 (76.9%) of 39 interviewees did not require any additional intervention. In the presenteeism interviews, workers who were determined not to need interventions were categorized into the following three groups:

#### 1. Workers who were able to solve work functioning impairment by themselves

Seven workers had high WFun scores due to new hires and changes in their job tasks. At the time of the interview, the situation was improved by becoming familiar with the work procedures. Five workers presented with work-functioning impairment due to a temporary increase in factory production. They had been able to cope with the musculoskeletal pain associated with the workload by stretching and improving their work practices to prevent it from becoming chronic. At the time of the survey, three workers were suffering from health problems (hay fever, common cold, and urticaria), but their symptoms had improved at the time of the interview by taking over-the-counter medication. Two workers had work dysfunction due to burdens in their private lives, such as death in the family and birth of a child.

#### 2. Workers whose problems were solved with support from their family physician, supervisors, or colleagues

Two workers received proper treatment to improve their symptoms (cataracts and numbness in upper limbs due to car accident). Four workers had improved their situation with help from their supervisors, which included reducing manpower shortages, improving work appropriateness, and sharing information on issues. Four workers were able to share workloads and improve relationships in the organization with support from their colleagues.

#### 3. Workers who did not have significant presenteeism

Three workers did not read the questionnaire carefully and answered it haphazardly, resulting in higher WFun scores. At the time of the interview, they had no memory of completing the questionnaire and no productivity loss.

## Conclusion and implications

We conducted interviews with workers based on presenteeism surveys and identified various occupational health issues categorized to four types. Conducting presenteeism interviews may allow us to identify health-related workplace issues from a new perspective than previous occupational health approaches. According to the past health records in this workplace, 33 of 36 workers with presenteeism had not been interviewed by the occupational health practitioners in the past 3 years. The presenteeism interviews uncovered new workplace issues in most cases.

We found that the medical checkups and the presenteeism survey seemed to find workers with problems from different perspectives. Based on the results of the health checkups at the same time as our survey, there were 21 workers (3.5% of the 607 respondents in the presenteeism questionnaire) who we judged to need the interview with occupational physician to consider work restrictions. They were all in 565 non-interviewees (WFun <21). This result was consistent with a previous study that found a weak association between presenteeism loss and health risk calculated using health checkups^9)^. This means that if we had not conducted the presenteeism interviews, we could not have found all of the cases. This supported the value of implementing presenteeism interviews.

We were able to understand the causes of maladaptation between workers and their work. These findings provided several basic suggestions for improving occupational health.

In addition, the attempt to understand the background of presenteeism contributed to the improvement of issues that affects productivity and other business perspectives. The current survey revealed several important issues, such as harassment and inadequate training opportunities for new employees.

There are three limitations of the current survey. First, the survey was conducted with a relatively small number of people at a single facility. Second, we were unable to control for subjective factors that might have influenced the occupational physicians’ judgments in the interviews. Third, there may have been selection bias. Some workers suffering from presenteeism may not have completed the questionnaire. Therefore, we may have missed other significant workplace issues.

In the future, it will be necessary to verify the effectiveness of the presenteeism interview by conducting further surveys with more workers in different industries.

## Acknowledgments

This survey was funded by JSPS KAKENHI Grant Number JP19K11682. We are grateful to Manami Katsuki, the occupational health nurse of the workplaces, for her support. We thank Benjamin Knight MSc., from Edanz (https://jp.edanz.com/ac) for editing a draft of this manuscript.

## Disclosure

Approval of the research protocol: This study was approved by the ethics committee of the University of Occupational and Environmental Health, Kitakyushu, Japan, and was conducted in full accordance with the World Medical Association Declaration of Helsinki. Informed consent: We explained the study protocol to subjects and obtained opt-out consent. Registry and the registration no. of the study/trial: N/A. Animal studies: N/A.

## Conflict of interest

YF received a personal fee from Sompo Health Support Inc. for the copyright on WFun. The other authors have no conflict of interest.

## Data accessibility

The data used in this survey are not publicly available. Please contact the authors for more information.

## Type of contribution

KM, YF, and TN designed the study. KS and KM conducted interviews and interpreted the results. KS and KM wrote the paper. YF, TN, and MN revised the paper. The authors all approved the final draft.
